# Enhanced Bacitracin Production by Systematically Engineering S-Adenosylmethionine Supply Modules in *Bacillus licheniformis*

**DOI:** 10.3389/fbioe.2020.00305

**Published:** 2020-04-07

**Authors:** Dongbo Cai, Bowen Zhang, Jiang Zhu, Haixia Xu, Pei Liu, Zhi Wang, Junhui Li, Zhifan Yang, Xin Ma, Shouwen Chen

**Affiliations:** ^1^State Key Laboratory of Biocatalysis and Enzyme Engineering, Environmental Microbial Technology Center of Hubei Province, College of Life Sciences, Hubei University, Wuhan, China; ^2^Key Laboratory of Fermentation Engineering (Ministry of Education), Hubei Provincial Key Laboratory of Industrial Microbiology, School of Food and Biological Engineering, Hubei University of Technology, Wuhan, China; ^3^Lifecome Biochemistry Co., Ltd., Nanping, China

**Keywords:** bacitracin, S-Adenosylmethionine, *Bacillus licheniformis*, methionine, metabolic engineering

## Abstract

Bacitracin is a broad-spectrum veterinary antibiotic that widely used in the fields of veterinary drug and feed additive. S-Adenosylmethionine (SAM) is a critical factor involved in many biochemical reactions, especially antibiotic production. However, whether SAM affects bacitracin synthesis is still unknown. Here, we want to analyze the relationship between SAM supply and bacitracin synthesis, and then metabolic engineering of SAM synthetic pathway for bacitracin production in *Bacillus licheniformis*. Firstly, our results implied that SAM exogenous addition benefited bacitracin production, which yield was increased by 12.13% under the condition of 40 mg/L SAM addition. Then, SAM synthetases and Methionine (Met) synthetases from *B. licheniformis*, *Corynebacterium glutamicum*, and *Saccharomyces cerevisiae* were screened and overexpressed to improve SAM accumulation, and the combination of SAM synthetase from *S. cerevisiae* and Met synthetase from *B. licheniformis* showed the best performance, and 70.12% increase of intracellular SAM concentration (31.54 mg/L) and 13.08% increase of bacitraicn yield (839.54 U/mL) were achieved in resultant strain DW2-KE. Furthermore, Met transporters MetN and MetP were, respectively, identified as Met exporter and importer, and bacitracin yield was further increased by 5.94% to 889.42 U/mL via deleting *metN* and overexpressing *metP* in DW2-KE, attaining strain DW2-KENP. Finally, SAM nucleosidase gene *mtnN* and SAM decarboxylase gene *speD* were deleted to block SAM degradation pathways, and bacitracin yield of resultant strain DW2-KENPND reached 957.53 U/mL, increased by 28.97% compared to DW2. Collectively, this study demonstrated that SAM supply served as the critical role in bacitracin synthesis, and a promising strain *B. licheniformis* DW2-KENPND was attained for industrial production of bacitracin.

## Introduction

Bacitracin, an important peptide antibiotic that consists of 11 kinds of amino acids, is mainly produced by *B*. *licheniformis* and *Bacillus subtilis*. Bacitracin biosynthetase gene cluster in *B. licheniformis* spans over 45 kd of DNA, which containing four genes, *bacT*, *bacA*, *bacB* and *back*. Gene *bacT* encodes thioesterase, and the relationship between bacitracin synthesis with *bacT* is unclear. BacA is responsible for activating and polymerizing five kinds of amino acids (Ile, Cys, Leu, D-Glu, and Ile) at bacitracin tail, BacB and BacC are responsible for activating and polymerizing seven kinds of amino acids in heptapeptide loop (Wang D. et al., 2017). Bacitracin owns the advantages of wide antimicrobial spectrum, rapid excretion rate, low absorption of livestock and poultry, not easy to produce resistance etc., and it inhibits the synthesis of cell wall of most gram-positive and a few of gram-negative bacteria, thus, bacitracin is widely used as the feed additive in feed industry, however, the low-yield has hindered its application (Fang et al., 2017).

With the development of synthetic biology and metabolic engineering, many elements and strategies have been developed for antibiotic metabolic engineering breeding (Zhou et al., 2017). The Leu-responsive regulatory protein Lrp has been engineered to improve erythromycin and actinorhodin production in *Streptomyces* (Liu et al., 2017), and deletion of phosphorus metabolism regulator gene *phop* was proven to be beneficial for avermection, pimaricin, etc., production (Sola-Landa et al., 2003; Martin et al., 2017; Mendes et al., 2007). In addition, precursor supply served as the critical role in secondary metabolite synthesis (Wohlleben et al., 2012). Branched chain amino acid (BCAA) served as the critical role in erythromycin production, and strengthening BCAA supplies led to a 41% increase of erythromycin yield, via deleting *lrp* and overexpressing BCAA transporter SACE_5387 (Liu et al., 2017). Moreover, acety-CoA carboxylase was overexpressed to improve malonyl-CoA supply, which further led to a 43% increase of surfactin production, reached 13.37 g/L (Wang et al., 2019). In addition, efficient use of substrate is also critical for metabolite production. Previously, maltose ABC transporter MalEFG was strengthened to improve utilization rate of corn starch, resulting in a 3.3-fold increase of ivermectin production in *Streptomyces* (Li et al., 2010). Generally, S-adenosylmethionine (SAM) served as the critical role in antibiotic biosynthesis, and strengthening SAM supply has been proven as an efficient strategy for lincomycin, avermectin, novobiovin, cephalosporin, etc., production (Zhao et al., 2010). However, whether SAM supply affects bacitracin synthesis has not been investigated in *Bacillus*.

S-Adenosylmethionine is one of the most widely used cofactor for group transfer reactions that involved in various metabolic processes, and it serves as the main methyl donor for DNA methylation, protein and secondary metabolite syntheses (Ruan et al., 2019), and SAM-dependent methylation is also the critical step for metabolite production. In addition, SAM addition improves antibiotic synthesis might via activating the transcription of pathway-specific regulatory genes or increasing the auto-phosphorylation of regulatory kinase (Kim et al., 2003). Generally, SAM is synthesized from aspartate, which generated from glucose via glycolytic and tricarboxylic acid (TCA) cycle, and methionine (Met) serves as the direct precursor for SAM biosynthesis (Ruan et al., 2019). Recently, several strategies have been conducted to improve SAM production, including: (i) enhancing activity of SAM synthetase, (ii) deleting cystathionin–β-synthase, (iii) releasing the feedback inhibitions of SAM to SAM synthetase and methylenetetrahydrofolate reductase (Chu et al., 2013). In addition, SAM titer was significantly enhanced via coupling SAM synthetic pathway with TCA cycle in *Bacillus amyloliquefaciens* (Ruan et al., 2019).

*Bacillus licheniformis* DW2 is an industrial strain for bacitracin production (Cai et al., 2019b), and several metabolic engineering approaches have been developed to improve bacitracin production. The synthetic pathways of Lysine (Lys) and Ornithine (Orn) were strengthened, which led to 28.95 and 16.5% increases of bacitracin yields, respectively (Wu et al., 2019; Yu et al., 2019). BCAA transporters BrnQ and YdhG were engineered to improve intracellular BCAA accumulations for bacitraicn synthesis (Li et al., 2018; Zhu et al., 2018). Additionally, the main regulators in carbon, nitrogen and phosphorus metabolisms were engineered, which led to a 35.72% increase of bacitraicn yield (Cai et al., 2019b). Due to the importance of SAM supply on antibiotic synthesis, in this study, we want to improve bacitracin production via rewiring SAM synthetic pathways, including in SAM synthetic, degradation and Met transportation pathways. Our results demonstrated that SAM supply served as a critical role on bacitracin synthesis, and this study provided a promising strain for industrial production of bacitracin.

## Materials and Methods

### Strains, Plasmids and Cultivation Conditions

The strains and plasmids used in this research were listed in Table 1. *B. licheniformis* DW2 acted as the original strain for constructing recombinants (Zhu et al., 2019), and *Escherichia coli* DH5α served as the host for vector construction. The plasmid T_2_(2)-Ori was applied for gene deletion, promoter replacement and gene integration *in B. licheniformis*, pHY300PLK was applied for constructing gene expression vector. All primers used in this research were provided in Supplementary Table S1.

**TABLE 1:** The strains and plasmids used in this research.

Strains and plasmids	Relevant properties	Source of references
**Strains**		
*Escherichia coli* DH5α	*sup*E44 Δ*lac*U169 (f 80 *lacZ*ΔM15) *hsd* R17 *recA*1 *gyr*A96 *thi*1 *rel*A1	This study
*Bacillus licheniformis* DW2	Wide-type CCTCC M2011344	CCTCC
DW2/pHY-metK_Bl_	DW2 harboring SAM synthetase MetK expression vector pHY-metK_Bl_	This study
DW2/pHY-metK_Cg_	DW2 harboring SAM synthetase MetK_Cg_ expression vector pHY-metK_Cg_	This study
DW2/pHY-SAM2	DW2 harboring SAM synthetase SAM2 expression vector pHY-SAM2	This study
DW2/pHY-300	DW2 harboring pHY300PLK, as the control strain	This study
DW2-K	SAM synthetase SAM2 integrated overexpression strain, based on strain DW2	This study
DW2-K/pHY-metH_Bl_	DW2-K harboring Met synthetase MetH expression vector pHY-metH_Bl_	This study
DW2-K/pHY-metH_Cg_	DW2-K harboring Met synthetase MetH expression vector pHY-metH_Cg_	This study
DW2-K/pHY-Met6	DW2-K harboring Met synthetase Met6 expression vector pHY-Met6	This study
DW2-KE	Met synthetase MetH overexpression strain via promoter replacement, based on strain DW2-K	This study
DW2/pHY-metN	DW2 harboring Met transporter MetN expression vector pHY-metN	This study
DW2/pHY-metP	DW2 harboring Met transporter MetP expression vector pHY-metP	This study
DW2△metN	Deletion of *metN* in DW2	This study
DW2△metP	Deletion of *metP* in DW2	This study
DW2-KEN	Deletion of *metN* in DW2-KE	This study
DW2-KENP	Overexpression of *metP* in DW2-KEN	This study
DW2-KENP△mtnN	Deletion of *mtnN* in DW2-KENP	This study
DW2-KENP△speD	Deletion of *speD* in DW2-KENP	This study
DW2-KENPND	Deletion of *mtnN* and *speD* in DW2-KENP	This study
**Plasmids**		
pHY300PLK	*E. coli* and *B. s* shuttle vector; Amp^*r*^, Tet^*r*^	Lab collection
T_2_(2)-Ori	*Bacillus* knockout vector; Kan^*r*^	Lab collection
pHY-metK_Bl_	SAM synthetase MetK_Bl_ expression vector, based on pHY300PLK	This study
pHY-metK_Cg_	SAM synthetase MetK_Cg_ expression vector, based on pHY300PLK	This study
pHY-SAM2	SAM synthetase SAM2 expression vector, based on pHY300PLK	This study
pHY-MetH_Bl_	Met synthetase MetH_Bl_ expression vector, based on pHY300PLK	This study
pHY-metH_Cg_	Met synthetase MetH_Cg_ expression vector, based on pHY300PLK	This study
pHY-Met6	Met synthetase Met6 expression vector, based on pHY300PLK	This study
pHY-metN	Met transporter MetN expression vector, based on pHY300PLK	This study
pHY-metP	Met transporter MetP expression vector, based on pHY300PLK	This study
T_2_-:SAM2	T_2_(2)-Ori-*SAM2*(A+*SAM2*+B); to overexpress *SAM2*	This study
T_2_-PbacA-PmetH	T_2_(2)-Ori-*PmetH*(A+PbacA+B); to replace the promoter of *metH* by PbacA	This study
T2-metN	T_2_(2)-Ori-*metN*(A+B); to delete *metN*	This study
T2-metP	T_2_(2)-Ori-*metP*(A+B); to delete *metP*	This study
T_2_-PbacA-PmetP	T_2_(2)-Ori-*PmetP*(A+PbacA+B); to replace the promoter of *metP* by PbacA	This study
T2-mtnN	T_2_(2)-Ori-*mtnN*(A+B); to delete *mtnN*	This study
T2-speD	T_2_(2)-Ori-*speD*(A+B); to delete *speD*	This study

Luria-Bertani (LB) medium was served as the basic medium for strain cultivation, and corresponding antibiotics (20 mg/L kanamycin, 20 mg/L tetracycline or 50 mg/L ampicillin) were added when necessary. The seed culture was inoculated in a 250 mL flask containing 20 mL LB medium for 6 h, and transferred (1 mL) into bacitracin production medium (10% soybean meal, 4.5% corn starch, 0.1% (NH_4_)_2_SO_4_, 0.6% CaCO_3_, natural pH), and then cultivated at 37°C, 230 r/min for 48 h. In order to analyze the function roles of Met transporters MetN and MetP, ME medium (20 g/L glucose, 20 g/L sodium glutamate, 10 g/L sodium citrate, 7 g/L NH_4_Cl, 0.5 g/L K_2_HPO_4_ 3H_2_O, 0.5 g/L MgSO_4_ 7H_2_O, 0.04 g/L FeCl_3_*cdot*6H_2_O, 0.104 g/L MnSO_4_*cdot*H_2_O, 0.15 g/L CaCl_2_ 2H_2_O, pH 7.2) was applied (Birrer et al., 1994). All the fermentation experiments were repeated at least three times.

### Construction of Gene Expression Vector

The gene expression vector was constructed according to our previous research (Zhu et al., 2019), based on pHY300PLK, and SAM synthetase MetK from *B. licheniformis* expression vector pHY-metK_Bl_ was served as an example. Briefly, P43 promoter from *B. subtilis* 168, gene *metK* and *amyL* terminator from *B. licheniformis* DW2 were amplified by corresponding primers (Supplementary Table S1), and fused by Splicing Overlap Extension (SOE)-PCR. The fused fragment was inserted into pHY300PLK at restriction sites *Eco*RI/*Xba*I, diagnostic PCR and DNA sequence confirmed that gene expression vector was constructed successfully, named as pHY-metK_Bl_. Similarly, other gene expression vectors were attained by the same method.

### Gene Deletion in *B. licheniformis*

The method for gene deletion in *B. licheniformis* was referred to our previously reported research (Cai et al., 2019b), and Met transporter gene *metN* deletion strain was served as an example. In brief, the upstream and downstream homology arms of *metN* were amplified from *B. licheniformis* DW2, and fused by SOE-PCR. The fused fragment was inserted into T_2_(2)-Ori at restriction sites *Sac*I/*Xba*I, diagnostic PCR and DNA sequence confirmed that gene deletion vector was constructed successfully, named T2-metN. Then, T2-metN was transferred into *B. licheniformis* DW2 via electroporation, and the positive transformants were cultivated in LB medium with 20 mg/L kanamycin at 45°C, and sub-cultured for three generations. Then, transferred into LB medium and sub-cultured for six generations at 37°C. The gene *metN* deletion strain was attained via homologous double crossover, diagnostic PCR and DNA sequence confirmed that *metN* deletion strain was constructed successfully, named as DW2ΔmetN.

### Gene Integrated Expression in *B. licheniformis*

To construct the strain which overexpressing SAM synthetase SAM2 from *Saccharomyces cerevisiae*, gene *SAM2* mediated by P43 promoter was integrated into the chromosome of *B. licheniformis* DW2, following to previously reported protocol (Cai et al., 2018), diagnostic PCR and DNA sequence confirmed that the gene integrated overexpression strain was constructed successfully.

### Promoter Replacement in *B. licheniformis*

The method for gene promoter replacement in *B. licheniformis* was referred to our previously reported research (Wu et al., 2019), and the procedure for promoter of Met synthetase MetH replaced by PbacA, a strong promoter that has been proven in our previous research (Shi et al., 2019), was served as an example. Briefly, the upstream and downstream homology arms of PmetH, promoter PbacA, were amplified to form T2-PbacA-PmetH, and promoter PmetH was replaced by PbacA via homologous double crossover, which procedure was the same as that of gene deletion.

### Analytical Methods

Bacitracin yield was measured by Agilent 1260 high performance liquid chromatography (HPLC), equipped with C18 column (ZORBAX SB-C18). Cell biomass was determined by dilution coating method (Cai et al., 2019a). SAM concentration was determined by HPLC (Ruan et al., 2019), and concentrations of intracellular and extracellular amino acids were measured by gas chromatography (GC), according to our previously reported research (Cai et al., 2019b). The transcriptional levels of bacirtacin synthetase gene cluster were determined by RT-qPCR, and gene *16S rDNA* was served as the reference for data normalization (Cai et al., 2017).

### Statistical Analysis

All data were conducted to analyze the variance at *P* < 0.05 and *P* < 0.01, and a *t*-test was applied to compare the mean values using the software package Statistica 6.0, and bars represented the standard deviations, ^∗^*P* < 0.05; and ^∗∗^*P* < 0.01 indicated the significance levels between recombinant strains and control (Cai et al., 2018).

## Results

### Exogenous SAM Addition Benefited Bacitracin Production

**FIGURE 1:**
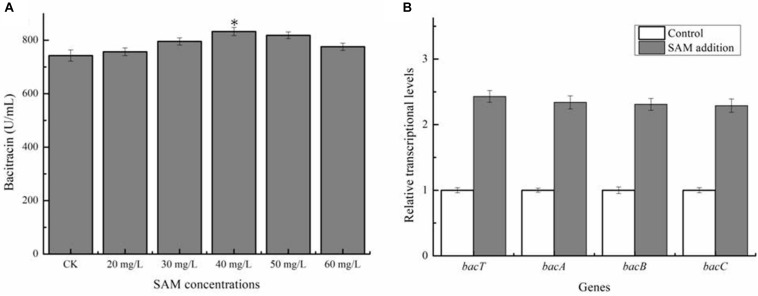
Effects of exogenous SAM addition on bacitracin production. **(A)** Effects of different concentrations of SAM (20, 30, 40, 50, and 60 mg/L) additions on bacitracin yield, **(B)** Effects of SAM addition on the transcriptional levels of bacitracin synthetase genes *bacT*, *bacA*, *bacB*, and *bacC*. ^∗^*P* < 0.05 and ^∗∗^*P* < 0.01 indicate the significance levels between recombinant strains and control strain.

Previously, SAM was proven to own the critical role in the syntheses numerous kinds of antibiotic (Zhao et al., 2010), however, whether SAM supply affect bacitracin synthesis has not been clarified in *Bacillus*. Here, different concentrations of SAM was, respectively, added into bacitracin production medium at 24 h (The beginning period of bacitracin synthesis), and our results in Figure 1A implied that exogenous SAM addition benefited bacitracin production, and the maximum bacitracin yield (832.45 U/mL) was attained under the condition of 40 mg/L SAM addition, increased by 12.13% compared to control (742.43 U/mL). Transcriptional levels of bacitracin synthetase genes *bacT*, *bacA*, *bacB* and *bacC* were measured under optimal condition, and our results implied that SAM addition improved transcription of bacitracin synthetase, increased by 1. 43-, 1. 34-, 1. 31-, and 1.29-fold, respectively (Figure 1B). In addition, our results demonstrated that SAM addition have no effects on cell biomass (Supplementary Figure S1). Moreover, the concentrations of intracellular SAM and Met were determined before (24 h) and after (36 h) SAM addition. Our results implied that the concentrations of intracellular SAM was 7.54 mg/L at 24 h, and which was increased to 17.29 mg/L at 30 h when SAM addition, increased by 53.69% compared to the control group (11.25 mg/L). While, Met concentrations showed no significant differences between two groups (Supplementary Figure S2). Taken together, these above results demonstrated that SAM served as the key role in bacitracin synthesis, and strengthening SAM supply was conducive to bacitracin production.

### Overexpressing SAM Synthetase Improved SAM Accumulation and Bacitracin Yield

**FIGURE 2:**
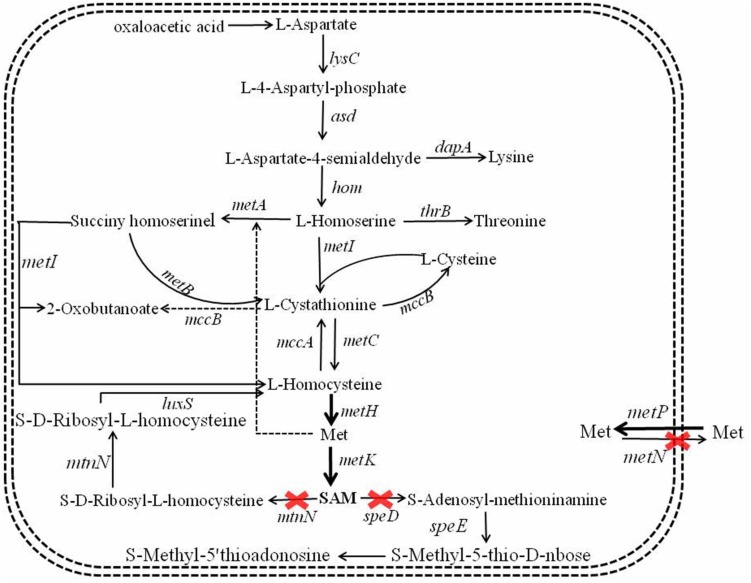
Metabolic engineering of SAM synthetic and degradation, Met transportation pathways for SAM accumulation and bacitracin synthesis in *B. licheniformis*.

S-Adenosylmethionine synthetase served as the key role in SAM formation from Met and ATP (Kanai et al., 2017), and overexpression of SAM synthetase benefited SAM production (Ruan et al., 2019). The SAM synthetic pathway of *B. licheniformis* was showed in Figure 2, here, SAM synthetases from *B. licheniformis*, *Corynebacterium glutamicum*, *S. cerevisiae* were strengthened in *B. licheniformis* DW2, attaining recombinant strains DW2/pHY-MetK_Bl_, DW2/pHY-MetK_Cg_ and DW2/pHY-SAM2, respectively.

**FIGURE 3:**
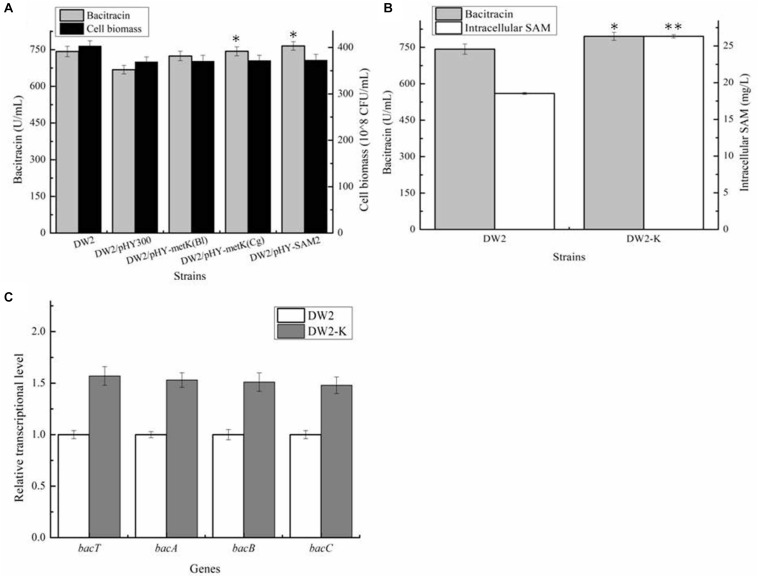
Effects of strengthening SAM synthetase expression on SAM accumulation and bacitracin production. **(A)** Effects of SAM synthetase overexpression on bacitracin yield and cell biomass, **(B)** Effects of integrated overexpression of SAM2 from *S. cerevisiae* on bacitraicn yield, cell biomass and SAM accumulation, **(C)** Effects of SAM2 overexpression on the transcriptional levels of bacitracin synthetase genes *bacT*, *bacA*, *bacB*, and *bacC*. ^∗^*P* < 0.05 and ^∗∗^*P* < 0.01 indicate the significance levels between recombinant strains and control strain.

Then, all these recombinant strains were cultivated in bacitracin production medium, as well as controls DW2 and DW2/pHY300. Based on our results of Figure 3A, bacitracin yields produced by DW2/pHY-MetK_Bl_, DW2/pHY-MetK_Cg_ and DW2/pHY-SAM2 were 724.32 U/mL, 743.42 U/mL and 765.31 U/mL, increased by 8.40, 11.24, and 14.51% compared with that of DW2/pHY300 (668.27 U/mL). In addition, cell biomasses of these strains showed no significant differences, and bacitracin produced by per cell were 1.95^∗^10^–8^ U/CFU, 2.00^∗^10^–8^ U/CFU and 2.05 U^∗^10^–8^/CFU, 7.97%, 10.46 and 13.43% higher than that of DW2/pHY300 (1.81^∗^10^–8^ U/CFU), respectively. Furthermore, *SAM2* expression cassette was inserted into chromosome of DW2, attaining SAM2 integrated overexpression strain DW2-K. Based on the results of Figure 3B, bacitracin yield of DW2-K reached 795.42 U/mL, increased by 7.14% compared to DW2 (742.43 U/mL). The bacitracin produced by per cell was 1.98^∗^10^–8^ U/CFU, increased by 6.30% (1.84^∗^10^–8^ U/CFU). The concentration of intracellular SAM (36 h) in DW2-K was 26.32 mg/L, 41.96% higher than that of DW2 (18.54 mg/L). Additionally, transcriptional levels of genes *bacT*, *bacA*, *bacB and bacC* were also increased significantly (Figure 3C), as the increase of SAM accumulation in DW2-K.

### Strengthening Precursor Met Supply Benefited SAM Accumulation and Bacitracin Production

**FIGURE 4:**
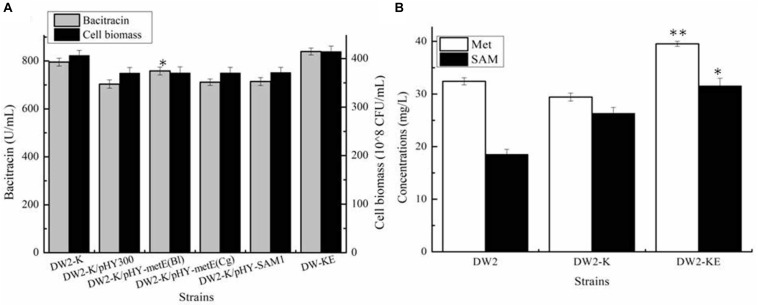
Effects of methionine synthetase overexpression on bacitracin production and cell biomass. **(A)** Bacitracin yield and cell biomass, **(B)** The concentrations of intracellular SAM and Met. ^∗^*P* < 0.05 and ^∗∗^*P* < 0.01 indicate the significance levels between recombinant strains and control strain.

Acting as the precursor for SAM synthesis, Met supply might affect the synthetic efficiency of SAM, which further affect bacitracin production. Here, Met synthetases from *B. licheniformis* (MetH), *C. glutamicum* (MetH), *S. cerevisiae* (Met6) were strengthened in DW2-K, attaining strains DW2-K/pHY-MetH_Bl_, DW2-K/pHY-MetH_Cg_ and DW2-K/pHY-Met6, respectively. Our results implied that overexpression of MetH from *B. licheniformis* benefited bacitracin synthesis, however, strengthening Met synthases from *C. glutamicum* and *S. cerevisiae* has no positive effect on bacitracin production. After that, the promoter of *metH* of DW2-K was replaced by bacitracin synthetase cluster promoter PbacA, a proven strong promoter that has been confirmed in our previous research (Shi et al., 2019), attaining MetH overexpression strain DW2-KE. Based on our results, bacitracin produced by DW2-KE reached 839.54 U/mL, increased by 5.55 and 13.08% compared to DW2-K and DW2, respectively. Bacitracin produced by per cell was 2.03^∗^10^–8^ U/mL, increased by 11.89 and 3.51% (Figure 4A). In addition, the intracellular Met and SAM concentrations (36 h) were 39.54 mg/L and 31.54 mg/L, increased by 34.40 and 19.83% compared with those of DW2-K (29.42 mg/L and 26.32 mg/L), respectively (Figure 4B). Taken together, all these above results demonstrated that strengthening Met synthetic pathway benefited SAM accumulation and bacitracin production.

### Identification and Engineering Met Transporters MetN and MetP Improved SAM Supply and Bacitracin Yield

Apart from metabolic pathway, engineering transportation pathway is also regarded as an efficient approach to improve target metabolite production. Previously, amino acid transporters LysE, BrnFE, and TcyP have been identified and engineered for Lys, Val and Cys production (Chen et al., 2015; Ohtsu et al., 2015; Dong et al., 2016), and deleting Lys transporter LysE and BCAA permease YhdG were also proven to be beneficial for bacitracin syntheses (Li et al., 2018; Wu et al., 2019). Here, MetN and MetP were annotated as Met transporters in *B. licheniformis* DW2, however, transportation modules of them have not been clarified, nor their roles on bacitracin production.

**FIGURE 5:**
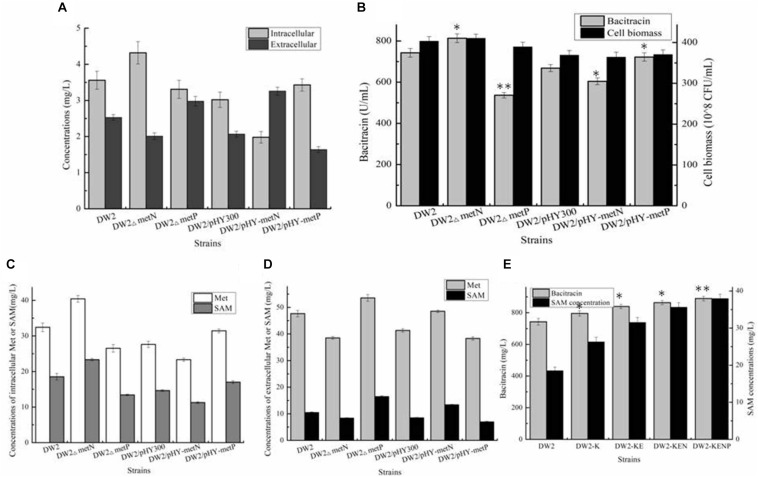
Identifying and engineering Met transporters for enhancement production of bacitracin. **(A)** The extracellular and intracellular of Met in ME medium, **(B)** Effects of deletion and overexpression of *metN* and *metP* on bacitracin production and cell biomass, **(C)** The concentrations of intracellular Met and SAM, **(D)** The concentrations of extracellular Met and SAM, **(E)** Effects of *metN* deletion and *metP* overexpression on bacitracin yields and intracellular SAM concentrations. ^∗^*P* < 0.05 and ^∗∗^*P* < 0.01 indicate the significance levels between recombinant strains and control strain.

To elucidate the transportation modes of these two transporters, genes *metN* and *metP* were deleted and overexpressed in DW2, attaining DW2△metN, DW2△metP, DW2/pHY-MetN and DW2/pHY-MetP, respectively. Then, these strains were cultivated in ME medium, and the concentrations of intracellular and extracellular Met were measured after 24 h cultivation. Based on the results of Figure 5A, strengthening MetP expression benefited Met import, while the concentration of intracellular Met was dropped significantly in MetN overexpression strain, and vice versa. Collectively, these above results suggested that MetN and MetP might act as the Met exporter and importer in *B. licheniformis* DW2, respectively.

Then, all these strains were cultivated in bacitracin production medium, and further results implied that *metN* deletion and *metP* overexpression benefited bacitracin production, which yields were increased by 9.55 and 8.05%, meanwhile, bacitracin yields were decreased by 9.58 and 27.73% in *metN* overexpression and *metP* deletion strains, compared to DW2 and DW2/pHY300, respectively (Figure 5B). Moreover, the intracellular Met concentrations were increased by 24.71 and 13.71% in DW2△metN and DW2/pHY-MetP, which concentrations were dropped by 18.17 and 15.59% in DW2△metP and DW2/pHY-MetN, indicated that *metN* deletion and MetP overexpression benefited intracellular Met accumulation (Figures 5C,D). Since Met served as the critical role in SAM synthesis and accumulation, the transporters MetN and MetP were confirmed to act as Met exporter and importer, respectively.

To further improve bacitracin synthesis capability of DW2-KE, gene *metN* was deleted to attain DW2-KEN, and *metP* was further overexpressed via promoter replacement, attaining strain DW2-KENP. Bacitracin fermentation results implied that 889.42 U/mL bacitracin was produced by DW2-KENP, increased by 19.80 and 5.94%, compared to DW2 and DW2-KE, and the specific bacitracin yield of DW2-KENP were increased by 20.31 and 5.71%, respectively. Meanwhile, the intracellular SAM concentration was increased to 37.98 mg/L, increased by 104.85 and 20.42%, respectively (Figure 5E).

### Blocking SAM Degradation Pathways Improved Bacitracin Synthesis

**FIGURE 6:**
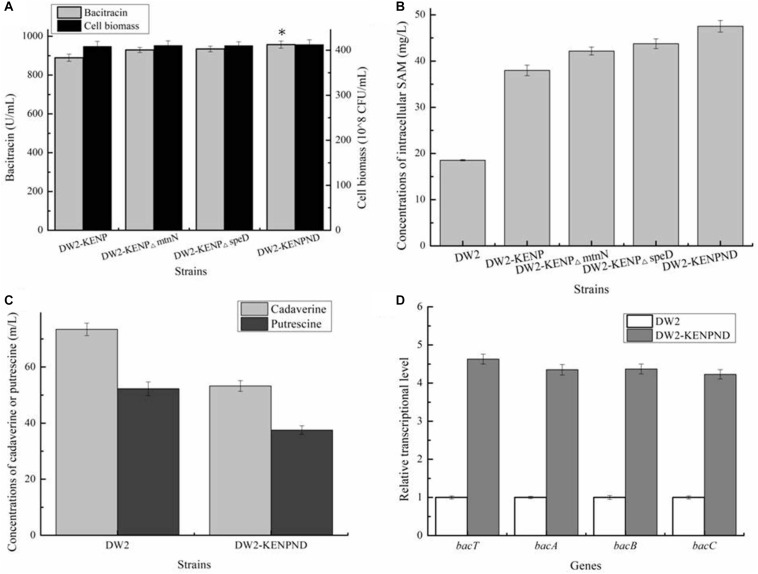
Effects of *mtnN* and *speD* deletion on bacitraicn production. **(A)** Bacitracin yield and cell biomass, **(B)** The concentration of intracellular SAM, **(C)** The concentrations of cadaverine and putrescine, **(D)** Transcriptional level analysis. ^∗^*P* < 0.05 and ^∗∗^*P* < 0.01 indicate the significance levels between recombinant strains and control strain.

In the above work, although concentration of intracellular SAM was enhanced significantly, the accumulated SAM might also be transformed into byproducts S-D-Ribosyl-L-homocysteine and S-Adenosyl-methioninamine, under the catalyses of SAM nucleosidase MtnN and SAM decarboxylase SpeD, respectively, which was not conducive to SAM accumulation. Here, genes *mtnN* and *speD* were deleted in DW2-KENP, resulting in DW2-KENP△mtnN and DW2-KENP△speD, respectively. As shown in Figure 6, deletion of *mtnN* and *speD* were beneficial for intracellular SAM accumulation, and bacitracin yields were also enhanced in gene deletion strains. Furthermore, the strain DW2-KENPND was attained via deleting *mtnN* and *speD* simultaneously, and bacitracin yield of DW2-KENPND reached 957.25 U/mL, increased by 28.93 and 7.63% compared to DW2 and DW2-KENP, respectively (Figure 6A). The intracellular SAM concentration of DW2-KENPND was 47.53 mg/L, increased by 1.56-fold compared to DW2 (Figure 6B). In addition, the harmful by-products during bacitraicn production, cadaverine and putrescine, produced by DW2-KENPND were 53.24and 37.52 mg/L, decreased by 27.50 and 37.04% compared with those of DW2 (73.43 mg/L and 52.25 mg/L), as the weakened expression of amino acid decarboxylase (Figure 6C). Moreover, the transcriptional levels of *bacT*, *bacA*, *bacB* and *bacC* were increased by 3. 63-, 3. 35-, 3. 37-, and 3.23-fold, respectively (Figure 6D).

### The Fermentation Processes of *B. licheniformis* DW2 and DW2-KENPND

**FIGURE 7:**
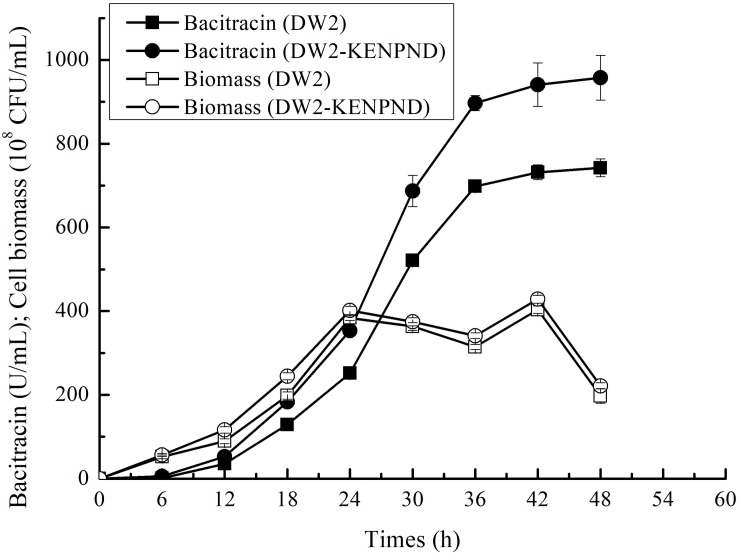
The fermentation process curves of *B. licheniformis* DW2 and DW2-KENPND.

Furthermore, the fermentation curves of *B. licheniformis* DW2 and DW2-KENPND were measured, and cell biomass and bacitracin yields were determined during fermentation process. Based on the results of Figure 7, bacitracin was synthesized from 12 h, and bacitracin yields of DW2-KENPND were higher than those of DW2 throughout the whole fermentation process, and the maximum yield produced by DW2-KENPND reached 957.53 U/mL, increased by 28.97%. In addition, the maximum cell biomass of DW2-KENPND was 428.54^∗^10^8^ CFU/mL, increased by 6.22% compared to DW2 (403.43^∗^10^8^ CFU/mL). The bacitracin produced by per cell was 2.23^∗^10^–8^ U/CFU, 21.42% higher than that of DW2 (1.84^∗^10^–8^U/CFU).

## Discussion

Bacitracin is a widely used veterinary antibiotic that mainly produced by *Bacillus*, and low production efficiency limits its promotion and application. SAM is an important cofactor in cell metabolism, which serves as the critical roles in syntheses of multiple antibiotics (Zhao et al., 2010). However, whether SAM affects antibiotic synthesis in *Bacillus*, especially for bacitracin, is still unknown. In this study, our results confirmed that strengthening intracellular SAM accumulation was beneficial for bacitracin production, and a metabolic engineered strain *B. licheniformis* DW2-KENPND with bacitracin yield of 957.53 U/mL was attained, via rewiring SAM synthetic and Met transportation pathways.

Acting as a critical methyl donor, SAM serves as the important role in the biosynthesis of multiple antibiotics (Zhang et al., 2008), in addition, SAM could increase the transcription of pathway-specific regulatory genes or auto-phosphorylation of regulatory kinase (Berney et al., 2015), therefore, strengthening SAM supply has been proven to be beneficial for production of lincomycin (Pang et al., 2015; Xu et al., 2018), avermectin (Tian et al., 2017), erythromycin (Wang et al., 2007), actinorhodin (Kim et al., 2003), etc. Moreover, SAM is not only involved in the transfer of sulfenyl and ribosyl groups, but also regulates intracellular metabolites that involved in primary and second metabolisms (Ruan et al., 2019). In this research, bacitracin is produced by Non-Ribosomal Polypeptide Synthetases (NRPS) in *B. licheniformis* (Rietkotter et al., 2008), different from those of above antibiotics (Polyketide Synthase) that which were mainly produced by *Streptomyces*, moreover, our results implied that strengthening SAM supply increased the transcription of bacitracin synthetase gene cluster, and transcriptional levels of genes *bacT*, *bacA*, *bacB*, and *bacC* were enhanced all under the condition of SAM addition. In addition, the transcriptional level of regulator gene *abrB* was decreased by 32.54% (Supplementary Figure S3). Since regulator AbrB has been proven as the negative regulator of bacitracin synthetase cluster *bacTABC* (Wang D. et al., 2017), our results suggested that additional SAM attenuated the inhibitory effect of AbrB on bacitracin synthase, and then improved bacitracin synthesis. Nevertheless, more work needs to be done to resolve the relationship between SAM and bacitracin synthetase.

Based on the previous researches, Met yield produced by microbial is generally at a high level (Huang et al., 2018), while SAM yield is quite low (Chu et al., 2013; Wendisch, 2019), indicating the low efficiency of SAM synthetic and transportation capabilities. Here, several SAM synthetases from *B. licheniformis*, *C. glutamicum* and *S. cerevisiae* were tested, and SAM2 from *S. cerevisiae* showed the best performances on SAM accumulation and bacitracin synthesis, consistent with the previous results of our group (Ruan et al., 2019). Along with developments of metabolic engineering and synthetic biology, various strategies have been conducted to improve SAM accumulation, including in strengthening SAM synthetic pathways (Zhang et al., 2008), coupling with TCA cycle (Ruan et al., 2019), increasing ATP level (Kanai et al., 2017), etc. In this research, the SAM synthetic and degradation pathways, Met transportation pathways were engineered to improve intracellular SAM accumulation for bacitracin production, and SAM concentration was increased to 47.53 mg/L by 1.56-fold, which led to a 28.97% increase of bacitracin yield. Based on the previous researches, the corresponding SAM transporters have been identified in *E. coli*, *Streptomyces coelicolor* etc., (Lee et al., 2012; Yang et al., 2014; Husna et al., 2018), also, the intracellular SAM concentration at 30 h was increased by 53.69% after SAM addition, suggested that additional SAM could be imported by amino acid transporter, thus, we suggested that SAM transporter is also present in *B. licheniformis* DW2, although it has not been identified or annotated. In addition, SAM decarboxylase gene *speD* was deleted to block synthetic pathway of byproduct, meantime, based on our results, two other harmful by-products, cadaverine and putrescine (Li et al., 2019), were also decreased by 27.50 and 37.04%, due to the weakening of amino acid decarboxylase (Figure 6C), and this result was positively correlated with our previous research (Wu et al., 2019). Furthermore, since SAM synthetase catalyzed the formation of SAM from Met and ATP, as well as the critical role of ATP supply in physiological metabolism (Cai et al., 2018), the ATP pool of DW2-KENPND should be enhanced in our future work.

Engineering transporter has been proven as an efficiency tactic for enhancement production of metabolite. Overexpression of ABC transporter AvtAB benefited avermectin secretion, and further led to the 50% increase of avermectin production (Qiu et al., 2011). Lys transporter LysE has been overexpressed to improve Lys production in *C. glutamicum* (Dong et al., 2016). However, bacitracin transporter in *Bacillus* has not been identified until now, although much work has been done on bacitracin microbial breeding and regulation (Fang et al., 2017; Wang D. et al., 2017; Shu et al., 2018). Here, two Met transporters MetN and MetP were screened, identified and engineered for bacitracin production at the first time. Based on our results, transporter MetN acted as Met exporter and MetP functioned as Met importer, and both of them served as the critical roles in Met distribution and bacitracin synthesis. Since soybean meal contains Met (Wang Q. et al., 2017), overexpression of MetP improved the accumulation of intracellular Met, which further benefit bacitracin production. Meantime, several other amino acid transporters (YdhG, BrnQ, and LysE) have also been engineered for bacitracin production (Li et al., 2018; Zhu et al., 2018; Wu et al., 2019), indicated that engineering amino acid transporter was an efficient strategy for bacitraicn production. Similar to Met transportation, several other amino acids also have multiple corresponding transporters. Such as, transporters YdhG, BrnQ, YvbW, and BraB were responsible for BCAAs transportation (Cai et al., 2019b), LysE, LysP and YvsH for lysine transportation (Wu et al., 2019), TcyP and YdeD for cysteine transportation (Ohtsu et al., 2015). The synergy of multiple transporters benefited the relative stability of intracellular and extracellular amino acids.

## Conclusion

S-Adenosylmethionine served as the critical role in antibiotic production, however, whether it affects bacitracin synthesis is still unknown. Here, our results confirmed that exogenous SAM addition benefited bacitracin production, and a metabolic engineered strain *B. licheniformis* DW2-KENPND was attained via rewiring SAM synthetic and degradation, Met transportation pathways. Based on our results, SAM synthetase SAM2 from *S. cerevisiae* and Met synthetase MetH from *B. licheniformis* showed the best performance on SAM accumulation and bacitracin syntheses, and Met transporters MetN and MetP were identified as Met exporter and importer in *B. licheniformis* DW2, respectively. Finally, The concentration of intracellular SAM of DW2-KENPND was 47.53 U/mL, increased by 1.56-fold compared to DW2, and bacitracin yield reached 957.53 U/mL, increased by 28.97%. Taken together, this research demonstrated that SAM served as the critical role in bacitraicn synthesis, and a promising strain *B. licheniformis* DW2-KENPND was attained for industrial production of bacitracin.

## Data Availability Statement

The datasets generated for this study are available on request to the corresponding author.

## Author Contributions

DC and SC designed the study. DC, BZ, JZ, and HX carried out the molecular biology studies and construction of engineering strains. DC, BZ, JZ, and PL carried out the fermentation studies. DC, ZW, JL, ZY, XM, and SC analyzed the data and wrote the manuscript. All authors read and approved the final manuscript.

## Conflict of Interest

JL was employed by Lifecome Biochemistry Co., Ltd., in China. The remaining authors declare that the research was conducted in the absence of any commercial or financial relationships that could be constructed as a potential conflict of interest.

## Abbreviations

BCAAs: branched chain amino acid
GC: gas chromatography
HPLC: high performance liquid chromatography
Lys: lysine
Met: methionine
NRPS: non-ribosomal polypeptide synthetases
Orn: ornithine
SAM: S-Adenosylmethionine.
