# Supplementary material for: Enhanced Bacitracin Production by Systematically Engineering S-Adenosylmethionine Supply Modules in Bacillus licheniformis
Source: Front Bioeng Biotechnol. 2020 Apr 7;8:305. doi: 10.3389/fbioe.2020.00305 (PMC7155746; doi:10.3389/fbioe.2020.00305)

Enhanced bacitracin production by systematically engineering S-adenosylmethionine supply modules in *Bacillus licheniformis*

Running title: Enhancing bacitracin production in B. licheniformis

Dongbo Cai [a], Bowen Zhang [a], Jiang Zhu [a], Haixia Xu [a], Pei Liu [a], Zhi Wang [b], Junhui Li [c], Zhifan Yang [a*], Xin Ma [a], Shouwen Chen [a*]

[a] *State Key Laboratory of Biocatalysis and Enzyme Engineering, Environmental Microbial Technology Center of Hubei Province, College of Life Sciences, Hubei University, Wuhan, 430062, PR China*

[b] *Hubei Provincial Key Laboratory of Industrial Microbiology, Key Laboratory of Fermentation Engineering (Ministry of Education), School of food and biological engineering, Hubei University of Technology, Wuhan* 430068, *Hubei*, *China*

[c] *Lifecome Biochemistry Co. Ltd, Nanping, 353400, PR China*

*Corresponding author：Prof. Zhifan Yang and Shouwen Chen

Tel./fax.: +86 027-88666081.

E-mail address: sailyangzhf@hubu.edu.cn and mel212@126.com (S. Chen).

Postal address: 368 Youyi Avenue, Wuchang District, Wuhan 430062, Hubei, PR China

Table S1 The primers used in this research

Primer names	Sequences (from 3' to 5')
pHY-F	GTTTATTATCCATACCCTTAC
pHY-R	CAGATTTCGTGATGCTTGTC
T2-F	ATGTGATAACTCGGCGTA
T2-R	GCAAGCAGCAGATTACGC
P43-F	CGGAATTC TGATAGGTGGTATGTTTTCG
P43-R	GTGTACATTCCTCTCTTACCTA
SAM2-F	TAAGAGAGGAATGTACACATGTCCAAGAGCAAAACTTTCT
SAM2-R	TCCGTCCTCTCTGCTCTTTTAAAATTCCAATTTCTTTGGT
MetK$_{Bl}$-F	TAAGAGAGGAATGTACACATGAGCAAAAACCGTCGGTTA
MetK$_{Bl}$-R	TCCGTCCTCTCTGCTCTTTTAATTATTCTCCTAATGCATCTTTC
MetK$_{Cg}$-F	TAAGAGAGGAATGTACAC
MetK$_{Cg}$-R	TCCGTCCTCTCTGCTCTTT
TamyL-F	AAGAGCAGAGAGGACGGATTTC
TamyL-R	GCTCTAGAGCCGCAATAATGCCGTCGCACTG
SAM2-F1	CGGGATCCCGTGAGGCGATGGATGTTCT
SAM2-R1	CGAAAACATACCACCTATCATTGACGAACCGTATCCGC
SAM2-F2	GCGGATACGGTTCGTCAATGATAGGTGGTATGTTTTCG
SAM2-R2	TCTTACCGTTTGCTGAGTGCGCAATAATGCCGTCGCACTGGC
SAM2-F3	GCCAGTGCGACGGCATTATTGCGCACTCAGCAAACGGTAAGA
SAM2-R3	GCTCTAGAGCTGTCAAACGCTCCGGTGG
SAM2-KYF	AGCTTCAATGCTACCCAAGCAGC
SAM2-KYR	GCCTTGTCTGAAATACATATA
MetH$_{Bl}$-F	TAAGAGAGGAATGTACACATGACAAATGTAAAAACGAG
MetH$_{Bl}$-R	TCCGTCCTCTCTGCTCTTTTAATTAAACAGTCTGAGCGAGTTTG
MetH$_{Cg}$-F	TAAGAGAGGAATGTACACATGAGCCAGAACCGCATCAGGACC
MetH$_{Cg}$-R	TCCGTCCTCTCTGCTCTTTTTAGAACAGTTCCTTTGATGCAA
Met6-F	TAAGAGAGGAATGTACACATGGTTCAATCTGCTGTCTTA
Met6-R	TCCGTCCTCTCTGCTCTTTTTAATTCTTGTATTGTTCACGG
PmetH-F1	CGACTTTCCGGGAGCGACTTC
PmetH-R1	TGAATCTCGCCGAAATCGCAGGTTGTTTTCTCCTTTTCTTTC
PmetH-F2	GAAAGAAAAGGAGAAAACAACCTGCGATTTCGGCGAGATTCA
PmetH-R2	CAGCTGGTCATTGATGTTAGACATATAAAAATTCTCCTTTTTGAT
PmetH-F3	ATCAAAAAGGAGAATTTTTATATGTCTAACATCAATGACCAGCTG
PmetH-R3	CCCATCGGTTCGATCGTGCCTGA
PmetH-KYF	ATATGAGGTCGCTGATCCATATT
PmetH-KYR	CTGACGGCCGTCCTCGCAAGGGAA
metN-F1	GCTCTAGACACGAATCTGATTGACAA
metN-R1	TCTGTAATCGTTCCGTGCCCATGCCGATCTTCCTTT
metN-F2	AAAGGAAGATCGGCATGGGCACGGAACGATTACAGA
metN-R2	GCGAGCTCAAACTCAGCACAAGCGAACC
metN-KYF	TTAAACGAAATTACAACTGCTA
metN-KYR	TAGCCGTATGTGATCGCC

metP-F1	GCTCTAGACGGAGGTTCTGCTCTGTG
metP-R1	AACTCAGCACAAGCGAACGACGAGCCTTGCGATGTA
metP-F2	TACATCGCAAGGCTCGTCGTTCGCTTGTGCTGAGTT
metP-R2	GCGAGCTCTTGGCGTCAATGTCACCG
metP-KYF	CGGAGGTTCTGCTCTGTG
metP-KYR	ACCGCTATGCCGTTGTTG
metN-F	TAAGAGAGGAATGTACACATGATTACATTTGAAGGCGTTGAA
metN-R	TCCGTCCTCTCTGCTCTTTTAATTATACCTCCCTGATGCGAATG
metP-F	TAAGAGAGGAATGTACACATGTTTGAAAAACTGTTTCCAA
metP-R	TCCGTCCTCTCTGCTCTTTTACTCAGAAACAGAAATGACA
PmetPQ-F1	GCTCTAGATCAGGCACGGTTGAAGTA
PmetPQ-R1	TCTCGCCGAAATCGCAGGATGCTTCAGCGTTTCCTGT
PmetPQ-F2	ACAGGAAACGCTGAAGCATCCTGCGATTTCGGCGAGA
PmetPQ-R2	CTTCCCACAGCTGCTCCATATAAAAATTCTCCTTTTTGAT
PmetPQ-F3	ATCAAAAAGGAGAATTTTTATATGGAGCAGCTGTGGGAAG
PmetPQ-R3	GCGAGCTCGCCGTTTACATAGGCAAGG
PmetPQ-KYF	AGTGCCCTATGAAACCCG
PmetPQ-KYR	AGCGGCTTTGCTTCTTCA
speD-F1	GCTCTAGAGTTGACCTTGTGGCTGAT
speD-R1	AATCGGCAGCTACGTTCGGATCCCCCACAGTTCGGAGATA
speD-F2	TATCTCCGAACTGTGGGGGATCCGAACGTAGCTGCCGAT
speD-R2	GCGAGCTCCTTCCAGCACTTCCTTTAC
speD-KYF	GGAAGAATGCACGGCCTGGC
speD-KYR	CTCCTCTTTCTTCCGACACC
mtnN-F1	GCTCTAGACGGAAAGGGAGTCACAGG
mtnN-R1	TGATAGGAAACCTGTGCGATTGACTTTGCCGATGCC
mtnN-F2	GGCATCGGCAAAGTCAATCGCACAGGTTTCCTATCA
mtnN-R2	GCGAGCTCATCGCACCCTTCATTCCG
mtnN-KYF	CGGGTGACAAGGGAAATA
mtnN-KYR	TTATGTCCGTGCGGTTCT
RT-bacA-F	GTATCTGCCGATCGACCCTG
RT-bacA-R	TCACAATCGATTCCGTCCGC
RT-bacT-F	ACTATCAAATGAGCGGGGCG
RT-bacT-R	GGATTGGGCAAGGTATGCAG
RT-bacB-F	GGACGAATCAGGCGAGAGAG
RT-bacB-R	GGACGAATCAGGCGAGAGAG
RT-bacC-F	GGACGAATCAGGCGAGAGAG
RT-bacC-R	TGATCTTCCTTCGCCACCAC
RT-abrB-F	CTAGGACGCGTGGTGATTCC
RT-abrB-R	CCAATTTACCGCCTGCAAGT

Fig. S1 Effects of SAM addition on the cell growth of *B. licheniformis*.

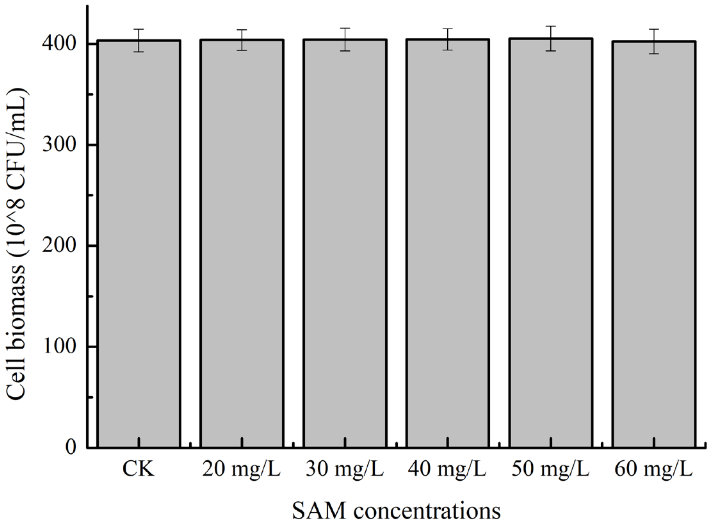

Fig. S2 The concentrations of intracellular Met and SAM before (24 h) and after (30 h) SAM addition.

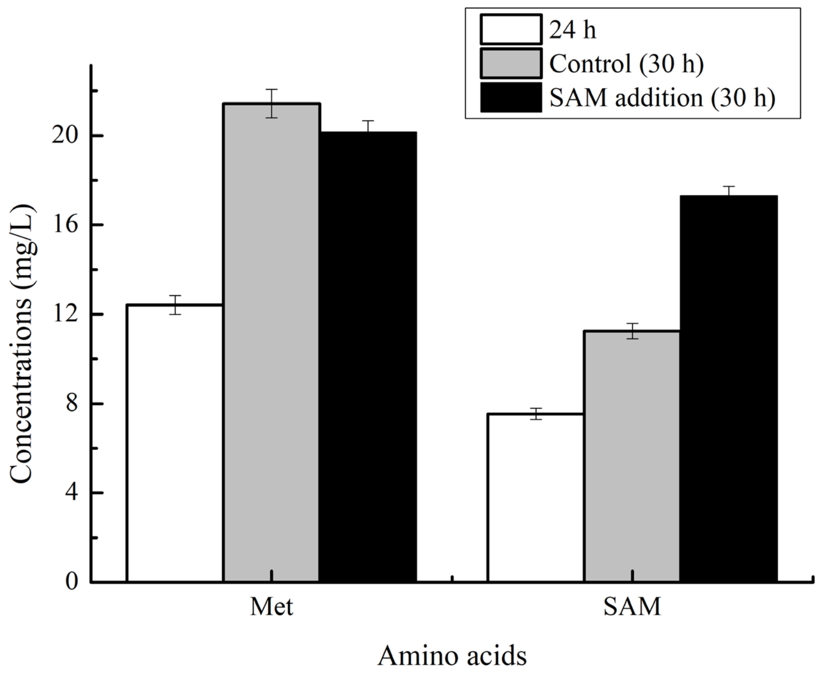

Fig.S3 Effects of SAM addition on the transcriptional level of regulator gene *abrB*.

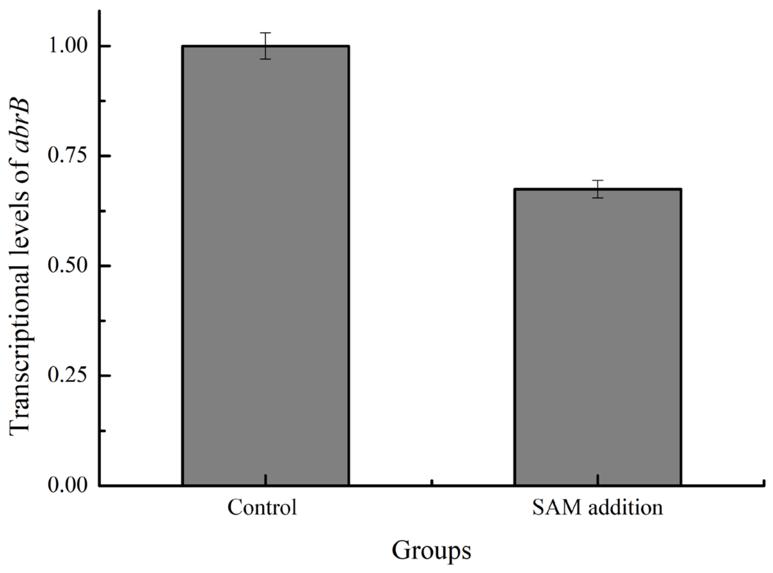

Supplement: Supplementary file 1 [file Data_Sheet_1.pdf]
